# Telomere length correlates with subtelomeric DNA methylation in long-term mindfulness practitioners

**DOI:** 10.1038/s41598-020-61241-6

**Published:** 2020-03-12

**Authors:** Maite Mendioroz, Marta Puebla-Guedea, Jesús Montero-Marín, Amaya Urdánoz-Casado, Idoia Blanco-Luquin, Miren Roldán, Alberto Labarga, Javier García-Campayo

**Affiliations:** 1 Neuroepigenetics Laboratory, Navarrabiomed Biomedical Research Center- UPNA-Navarra Institute for Health Research (IdiSNA), Pamplona, Navarra 31008 Spain; 2 Department of Neurology, Complejo Hospitalario de Navarra, Pamplona, Navarra 31008 Spain; 3 Instituto de Investigación Sanitaria de Aragón. Red de Investigación en Atención Primaria (REDIAPP), Zaragoza, Spain; 4 Department of Psychiatry, University of Oxford, Oxford, OX3 7JX UK; 5 Bioinformatics Unit, Navarrabiomed Biomedical Research Center - UPNA-Navarra Institute for Health Research (IdiSNA), Pamplona, Navarra 31008 Spain; 6 Miguel Servet University Hospital, University of Zaragoza, Zaragoza, Spain

**Keywords:** DNA methylation, Biomarkers, Biomarkers, DNA methylation

## Abstract

Mindfulness and meditation techniques have proven successful for the reduction of stress and improvement in general health. In addition, meditation is linked to longevity and longer telomere length, a proposed biomarker of human aging. Interestingly, DNA methylation changes have been described at specific subtelomeric regions in long-term meditators compared to controls. However, the molecular basis underlying these beneficial effects of meditation on human health still remains unclear. Here we show that DNA methylation levels, measured by the Infinium HumanMethylation450 BeadChip (Illumina) array, at specific subtelomeric regions containing *GPR31* and S*ERPINB9* genes were associated with telomere length in long-term meditators with a strong statistical trend when correcting for multiple testing. Notably, age showed no association with telomere length in the group of long-term meditators. These results may suggest that long-term meditation could be related to epigenetic mechanisms, in particular gene-specific DNA methylation changes at distinct subtelomeric regions.

## Introduction

In recent years, the use of mindfulness and meditation techniques have been increasing in western societies with the aim of reducing stress and improving overall health^1–4^. Mindfulness-based interventions have proved their beneficial effect on a number of medical and psychological conditions, including depression, anxiety, and immune disorders^5–8^. In addition, it has been proposed that meditation techniques could positively affect longevity^9,10^. In fact, intensive meditation training has been associated with an increase in telomerase activity^11^ and longer telomere length in blood cells^12–16^, which is considered a candidate biomarker of human aging.

Telomeres are DNA-protein complexes that protect the end of linear chromosomes from degradation, fusion, or DNA repair processes^17–19^. Telomeres shorten with every somatic cell division because of the end replication problem^19,20^, but this erosion is partially compensated by the action of telomerase, an enzyme complex that adds TTAGGG hexanucleotide repeats to the telomeric DNA^17,21^. However, telomerase expression levels in mammalian adult cells are not sufficient to preserve the original length of telomeres, resulting in the progressive shortening of chromosomes throughout life^20,22–24^. Telomere shortening has been associated with age-related conditions^25–28^, and telomere length is hypothesized to be a biomarker of aging and age-related morbidity^29–34^.

In line with other reports, our group previously observed a positive relationship between meditation practice and longer telomere length in peripheral blood cells^13^. Moreover, in a previous work to gain insight into the molecular mechanisms of meditation, we profiled genome-wide DNA methylation changes in long-term meditators and identified a set of 64 differentially methylated regions (DMRs), corresponding to 43 genes, when compared to controls^35^. Almost half of the DMRs (48.4%) were directly linked to common human diseases, including neurodegenerative disorders such as Alzheimer’s and Parkinson’s diseases. Surprisingly, up to 23.4% of these DMRs were found to be located at subtelomeric regions (hypergeometric test fold-enrichment = 1.3; p-value < 0.05). However, the importance of these epigenetic changes within the subtelomeric regions in meditators remains unclear.

Increasing evidence supports the idea that epigenetic changes in subtelomeric regions may be linked to telomere length. For instance, in blood cells from healthy middle-aged men it was found that CpG sites whose methylation level was associated with telomere length were significantly enriched at subtelomeric regions^36^. Most importantly, TET (Ten-eleven translocation) enzymes which are responsible of converting 5-methylcytosine to 5-hydroxymethylcytosine, have been proved crucial in telomere maintenance by modulating subtelomeric methylation levels^37^. Additional evidence on the potential role of DNA methylation in telomere homeostasis shows that mouse embryonic stem cells lacking DNA methyltransferases have decreased global methylation in subtelomeric regions and dramatically elongated telomeres^38^. On the contrary, telomere shortening to a critically short length leads to epigenetic defects at mammalian telomeres and subtelomeres, such as decreased subtelomeric DNA methylation^39,40^.

A number of pathological conditions support the possible involvement of subtelomeric DNA methylation in telomere length stability. Human mutations on the DNA methyltransferase *DNMT3B* [OMIM, * 602900], which cause immunodeficiency-centromeric instability-facial anomalies syndrome 1, are associated with subtelomeric global hypomethylation along with accelerated telomere shortening of fibroblasts^41,42^. Both direct and inverse correlation has been described for various types of human cancer between subtelomeric DNA methylation levels and telomere length^43,44^. To sum up, DNA methylation levels at subtelomeric regions might play a role in maintaining telomere length, among other mechanisms. However, the potential effect that subtelomeric DNA methylation levels may exert on telomere length and the underlying mechanisms remain unknown.

The aim of the present study was to better understand the relevance of subtelomeric DNA methylation changes in long-term meditators. To that end, we tested the association of telomere length with DNA methylation levels and age in long-term meditators and controls for this set of DMRs.

## Results

### Subjects characteristics

The general characteristics of subjects included in the study are shown in Table 1. Socio-demographic characteristics including age, gender, ethnicity, and BMI were equally distributed between groups. However, as expected, there were significant differences in the amount of practical experience of meditation and also in all the psychological health-related variables referred to below (see Methods section).

**Table 1:** Characteristics of study participants.

	Total sample (n = 34)	Controls (n = 17)	Meditators (n = 17)	p
Age^†^	49.47 (8.16)	48.59 (9.91)	50.35 (6.11)	0.536
Sex, Male^‡^	24 (70.60)	11 (64.70)	13 (76.50)	0.452
Education, University^‡^	18 (52.9%)	10 (58.8%)	8 (47.1%)	0.492
BMI^†^	24.32 (1.94)	23.73 (2.13)	24.91 (1.58)	0.078
Months of Practice^†^	106.47 (123.06)	0.00 (0.00)	212.94 (84.54)	<0.001
MAAS^†^ (range 1–6)	3.93 (0.60)	3.42 (0.25)	4.43 (0.38)	<0.001
FFMQ observing^†^ (range 8–40)	26.38 (2.76)	23.94 (1.03)	28.82 (1.43)	<0.001
FFMQ describing^†^ (range 8–40)	27.44 (2.06)	26.00 (1.12)	28.88 (1.76)	<0.001
FFMQ acting^†^ (range 8–40)	30.35 (2.89)	27.88 (1.50)	32.82 (1.43)	<0.001
FFMQ non judging^†^ (range 8–40)	29.29 (4.08)	26.41 (3.81)	32.18 (1.47)	<0.001
FFMQ non reacting^†^ (range 7–35)	24.00 (2.58)	22.88 (2.47)	25.12 (2.23)	0.009
HADS anxiety^†^ (range 0–21)	1.74 (1.52)	3.06 (0.90)	0.41 (0.51)	<0.001
HADS depression^†^ (range 0–21)	2.50 (2.02)	4.12 (1.05)	0.88 (1.32)	<0.001
CDRISC resilience^†^ (range 0–100)	27.91 (3.68)	24.53 (1.59)	31.29 (1.05)	<0.001
SHS happiness^†^ (range 4–28)	23.77 (3.65)	21.00 (1.80)	26.53 (2.83)	<0.001
SCS common humanity^†^ (range 2–8)	5.00 (1.16)	4.47 (1.01)	5.53 (1.07)	0.006
SCS mindfulness^†^ (range 2–8)	4.97 (1.53)	3.71 (0.77)	6.24 (0.90)	<0.001
SCS self-kindness^†^ (2–10)	4.88 (0.91)	4.47 (0.88)	5.29 (0.77)	0.007
SLWS satisfaction^†^ (range 5–35)	28.15 (2.36)	26.71 (2.20)	29.59 (1.50)	<0.001
AAQ-II avoidance^†^ (range 7–49)	18.65 (4.44)	22.41 (1.58)	14.88 (2.83)	<0.001

Figures represent means, standard deviations (^†^), and the p-value associated with a t contrast between the control group and the meditators group, except for sex and education, where the figures represent frequencies and percentages (^‡^) and the p-value associated with a χ2 contrast. Range indicates the bounds of the scales for each psychological variable.

### Relationship between DNA methylation levels and telomere length

From our genome-wide DNA methylation differential analysis between long-term MMs and controls^35^, we selected the group of 14 DMRs located in subtelomeric regions, involving chromosomes 2, 4, 5, 6, 7, 8, 16, 19, and 20. We define subtelomeric regions as those regions at either end of each chromosome with a length of 3000 kb. We selected this subtelomeric length as an operational definition in order to perform the bioinformatics analysis, since the exact bounderies for subtelomeres have not yet been well established. This group represents 23.4% of the total DMRs (hypergeometric test fold-enrichment = 1.3; p-value < 0.05) that were identified in long-term MMs compared to controls. Genes and DNA methylation levels for these regions are shown in Table 2.

**Table 2:** List of differentially methylated regions between long-term meditators and controls located in subtelomeric regions.

Gene alias	Chr	DMR-start	DMR-end	no.probes	p-value	beta.diff	Genomic Location
C8orf73/MROH6	chr8	144654887	144655484	4	0,002101627	−0,057729475	1stExon,5′UTR,TSS200,TSS1500
ERICH1-AS1	chr8	709576	709692	2	0,036605753	−0,059338436	
GPR31	chr6	167571172	167571803	5	0,023259942	−0,07099427	1stExon,TSS200,TSS1500
KBTBD11	chr8	1954777	1955196	4	3,90054E-08	−0,053	3′UTR
MADCAM1	chr19	495355	495737	2	0,001456746	−0,081370984	TSS1500
MLPH	chr2	238406108	238406478	3	0,003007912	−0,056124149	Body
MYL5,MFSD7	chr4	675137	675827	3	0,008481849	0,126013355	Body
PRR25	chr16	857454	857863	3	0,036715428	0,106034484	Body
RPS6KA2	chr6	166876490	166877038	7	0,000731957	−0,064536785	Body
RPS6KA2	chr6	167070053	167070616	3	0,009173941	0,081025691	Body
RUFY1	chr5	178986131	178986728	7	0,029542821	−0,063294921	TSS1500,Body,TSS200
SERPINB9	chr6	2891973	2892050	2	0,00012719	0,061725435	Body
SRXN1	chr20	633418	634604	4	0,004924204	−0,062058469	Body,TSS1500
UNCX	chr7	1266180	1267228	4	0,000393945	−0,114311707	
Intergenic	chr4	1514317	1514621	2	0,008962804	−0,086216947	

NA = not applicable, Chr = Chromosome, DMR = differentially methylated region, DMR-start = coordinates of the beginning of each DMR annotated by UCSC hg19 build, DMR-end = coordinates of the end of each DMR annotated by UCSC hg19 build, no.probes = total number of differentially methylated probes within each DMR, beta.diff = difference in average methylation between mindfulness-practitioners and controls for each DMR, UTR = untranslated region, TSS = transcription start site.

**Figure 1:**
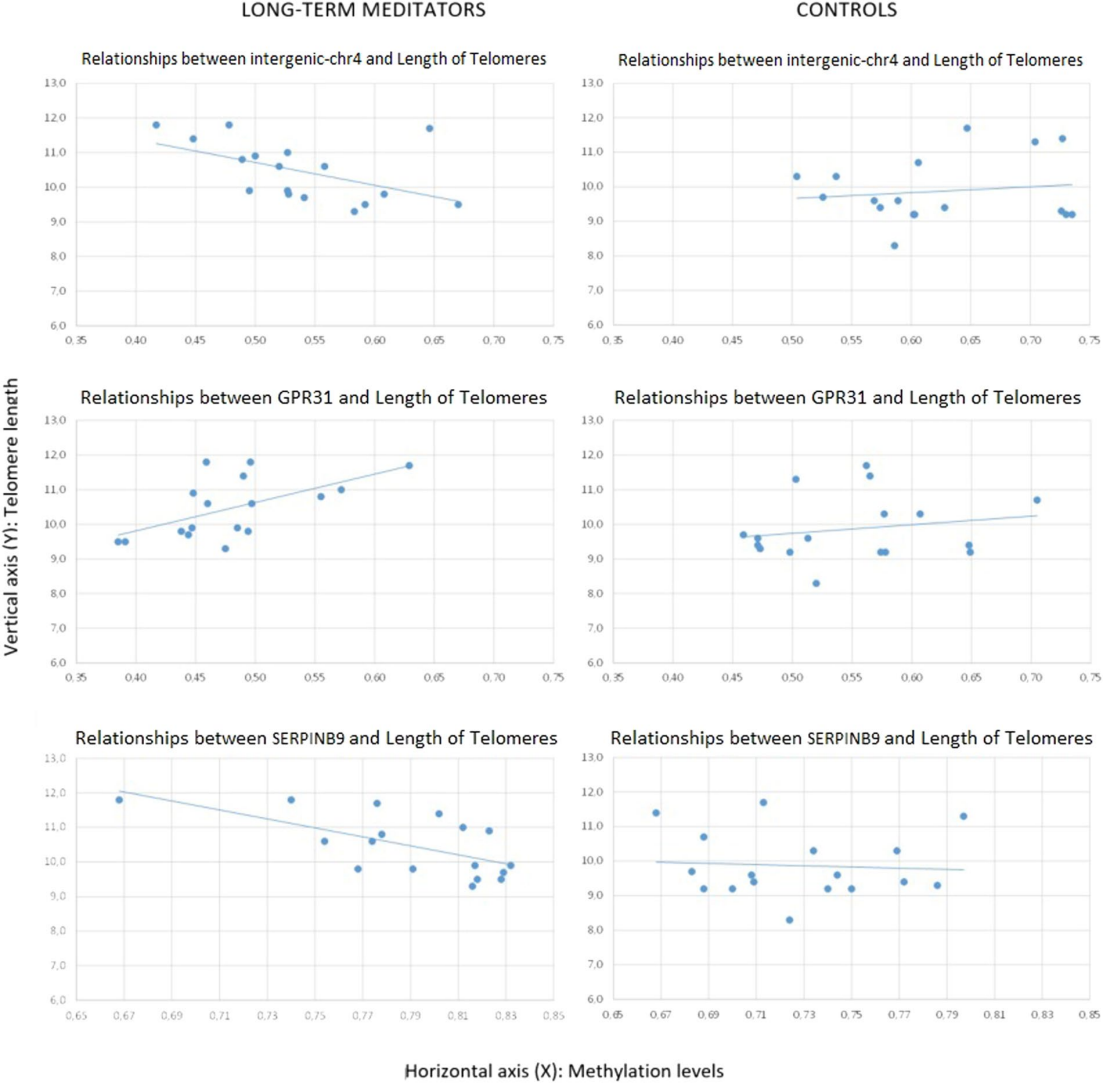
Relationships between intergenic (chr4: 1514317–1514621), *GPR31*, and *SERPINB9* DNA methylation levels and telomere length, according to group. The intergenic region (chr4: 1514317–1514621) is in the first row, *GPR31* in the second, and *SERPINB9* in the third. Long-term meditators are in the first column and controls are in the second. Methylation levels are represented in the horizontal axis (X), and telomeres length in the vertical axis (Y).

The raw differences between groups showed that telomere length was longer in meditators (Mn = 10.47; SD = 0.86) compared to controls (Mn = 9.87; SD = 0.94), without any statistical significance, but showing a strong trend (t = −1.94; df = 32; p = 0.061), with a moderately high ES (d = 0.67). However, the differences appeared to be significant (t = −3.44; df = 32; p = 0.002) after controlling for age, with a very high ES (d = 1.25). We then asked whether telomere length was correlated with gene-specific DNA methylation levels. In the group of long-term MMs, we found a positive raw correlation between telomere length and DNA methylation levels at the *GPR31* gene (r = 0.58, p = 0.014), whereas an inverse correlation was shown between telomere length and DNA methylation levels at two distinct loci: *SERPINB9* gene (r = −0.64, p = 0.006) and an intergenic CpG island within the subtelomeric region of chromosome 4 short arm (chr4:1514317–1514621, GRCh37/hg19 assembly) (r = −0.51, p = 0.036) (Fig. 1). After correction for age, the significant correlations remained (Table 3). To test for multiple comparisons, we draw at random 1000 sub-samples of 14 not differentially methylated regions within subtelomeric regions. The proportion of such random sets showing 3 or more hits with absolute r value greater than or equal to 0.51 (the minimum value for our selection) was 0.052 (>0.05). Therefore, the possibility of a false positive result cannot be completely excluded.

**Table 3:** Explanatory power of gene methylation on the length of long telomeres according to group.

	Meditators						Non- Meditators					
	Md	SD	r	R^2^	Beta	R^2^_y.12_	Md	SD	r	^R2^	Beta	R^2^_y.12_
						0.15						0.91***
Age					−0.39						−0.93***	
UNCX	0.592	0.135	−0.03	<0.01	0.03		0.706	0.050	−0.28	0.08	−0.11	
						0.41*						0.90***
Age					−0.38						−0.95***	
NA	0.537	0.067	−0.51*	0.26*	−0.51*		0.623	0.076	0.14	0.02	0.01	
						0.17						0.90***
Age					−0.46						−0.94***	
MADCAM1	0.414	0.064	0.03	<0.01	−0.18		0.496	0.060	0.37	0.14	0.04	
						0.45*						0.92***
Age					−0.33						−0.94***	
GPR31	0.480	0.061	0.58*	0.34*	0.55*		0.551	0.072	0.19	0.04	0.14	
						0.15						0.90***
Age					−0.39						−0.95***	
RPS6KA2	0.801	0.066	0.03	<0.01	0.05		0.720	0.053	−0.02	<0.01	−0.05	
						0.24						0.90***
Age					−0.40						−0.95***	
SFXN1	0.536	0.050	0.29	0.08	0.30		0.596	0.060	0.25	0.06	0.01	
						0.19						0.90***
Age					−0.28						−0.94***	
SRXN1	0.571	0.048	0.36	0.13	0.23		0.633	0.038	−0.15	0.02	−0.03	
						0.23						0.92***
Age					−0.42						−0.96***	
ERICH1_AS1	0.668	0.065	0.25	0.06	0.29		0.727	0.040	0.04	<0.01	0.13	
						0.15						0.90***
Age					−0.39						−0.95***	
C8orf73	0.252	0.043	−0.06	<0.01	−0.06		0.310	0.070	0.08	0.01	−0.01	
						0.16						0.90***
Age					−0.34						−0.95***	
MLPH	0.725	0.043	0.25	0.06	0.11		0.782	0.028	−0.29	0.08	0.01	
						0.20						0.90***
Age					−0.43						−0.95***	
KBTBD11	0.306	0.064	0.14	0.02	0.23		0.340	0.055	0.29	0.08	−0.01	
						0.40*						0.90***
Age					<0.01						−0.95***	
SERPINB9	0.790	0.042	−0.64**	0.40**	−0.64*		0.728	0.038	−0.07	0.01	−0.02	
						0.27						0.90***
Age					−0.31						−0.95***	
SSTR5	0.577	0.057	−0.43	0.18	−0.36		0.525	0.027	0.01	<0.01	0.03	
						0.16						0.91***
Age					−0.32						−0.96***	
MYL5	0.295	0.174	−0.29	0.08	−0.13		0.169	0.105	0.06	<0.01	−0.10	

Md = mean. SD = standard deviation. r = Pearson’s raw correlation between telomere length and gene methylation. R2 = bivariate determination coefficient (dependent variable: telomere length; independent variable: genes methylation). R2y.12 = multiple determination coefficient (dependent variable: telomere length; independent variables: age and gene methylation). Beta = standardized slope when using multiple regression models. *p < 0.05. **p < 0.01. ***p < 0.001.

**Figure 2:**
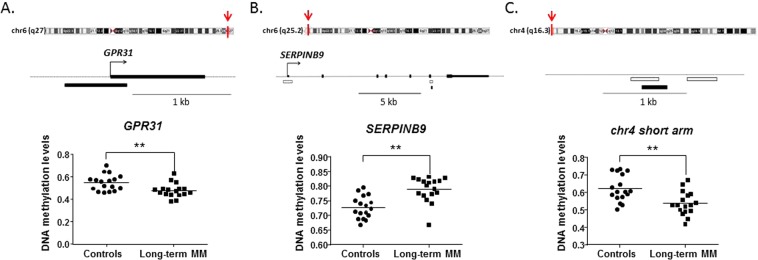
DNA methylation levels of differentially methylated regions (DMRs) identified in long-term meditators that were associated with telomere length. The upper track of each panel shows the subtelomeric position of mindfulness-related DMRs (human ideograms) and the genomic map of each region. Below each gene, white boxes denote CpG islands and black boxes represent DMRs. Dot-plot graphs show the results of the 450 K array (beta values) for GPR31 and SERPINB9 and the intergenic region (subtelomeric region at short arm of chr4).

Maps of these DMRs and differential DNA methylation levels between long-term MMs and controls are shown in Fig. 2. In contrast, no significant correlation was found between DNA methylation levels of any gene and telomere length in the control group, either before or after controlling for age (Table 3, Fig. 1).

### Relationship between age and telomere length

Since age is well known to be associated with progressive telomere shortening, we further checked the correlation between age and telomere length separately for each group. We found that age showed a strong inverse correlation with telomere length in the control group (r = −0.95, p < 0.001), as expected. In fact, the great explanatory power of multivariate models to explain telomere length in the control group was exclusively due to the intervention of age (Table 3). However, this inverse correlation between age and telomere length, although with a moderately low effect, was no longer seen to be significant for the meditation group (r = −0.38, p = 0.127), in which multivariate models were less powered to explain telomere length, and only some DNA methylation levels showed significant contributions (Table 3).

### Relationship between DNA methylation and mRNA expression

We measured *GPR31* and *SERPIN9* mRNA expression levels by real time quantitative PCR (RT-qPCR) in peripheral blood of long-term meditators compared to controls. Three control samples did not pass the RNA quality threshold (see Methods section) and so were not included in the experiments. Eventually, 17 long-term meditators were compared to 14 controls. After normalizing mRNA expression levels to the geometric mean of 2 housekeeping genes (*ACTB* & *TBP*), we found that blood expression was very low for *GPR31* (CTs >30), so no reliable differential analysis could be performed. In the case of *SERPIN9*, differential analysis showed no statistical significant difference in mRNA expression levels between meditators and controls (p = 0.493).

## Discussion

In this cross-sectional study, we have shown that telomere length is associated with DNA methylation levels in long-term MMs at three distinct subtelomeric regions, involving the *GPR31* and *SERPINB9* genes. Notably, telomere length did not correlate with age in the group of meditators, in contrast with the significant inverse correlation between telomere length and age in the comparison group.

Telomere length is involved in molecular and cellular senescence and has been proposed as a biomarker of human aging^29–34^. The progressive decrease in telomere length with age has long been known^22–24^. Short telomeres contribute to genomic instability that is permissive for cancer initiation and progression^17^. In addition, leukocyte telomere shortening has been associated with several age-related conditions, such as cardiovascular events, including stroke and myocardial infarction^25,26^, and cognitive performance^27^. In this scenario, telomerase-based therapies are emerging as novel approaches for the treatment of age-related diseases^45,46^.

While stressful life events and psychological stress have been consistently associated with leukocyte telomere erosion^47–50^, some healthy habits and behaviors have been related to longer telomere length or reported to reduce the rate of telomere shortening, e.g., physical activity and training^51–53^. In line with the previous factors, yoga practice and meditation are related to longer telomere length in blood cells^12,14–16^, as a previous work by our group has also shown^13^. Also in the case of cancer patients, such as distressed breast cancer survivors, mindfulness-based therapy is associated with telomere length maintenance^54^. Furthermore, it has been observed that mindfulness meditation leads to increased telomerase activity in PBMCs^15^. All this evidence suggests that some interventions may help to buffer the negative impact of stress on health and telomere length. A number of mechanisms have been postulated to mediate the effect of meditation on telomere stability, such as the modulation of the hypothalamic-pituitary-adrenal axis^55^ or reduction in oxidative stress and inflammatory pathways^20,56,57^. However, the biological substrate underlying this relationship remains largely unknown.

In the present study, differences between meditators and controls on telomere length were not significant before controlling for age, and thus did not replicate the raw findings from Alda *et al*.^13^. However, this could be due to the smaller sample size used that could reduce statistical power. In fact, results of the present study showed a trend very close to statistical significance, with a moderately high effect size, and differences appeared clearer and significant after controlling for age. Anyway, as expected, our study found a strong inverse correlation between age and telomere length in the control group. Interestingly, we observed that age was no longer related to telomere length in the group of long-term MMs. Due to the limited sample size, this finding may cautiously be interpreted as suggesting that long-term meditation may somewhat counteract the effect of biological aging on telomere length. This could be accounted for, at least in part, the fact that meditation significantly reduces stress and the biological correlates of stress on the human body^58,59^. Consistent with this idea, meditation has also been associated with longevity^60,61^. Anyhow, this is an exploratory study and the underlying molecular changes still need further investigation. This work will guide future research using more powered designs to control for possible errors.

Remarkably, we also found that telomere length was associated with gene-specific subtelomeric DNA methylation changes in the group of long-term MMs. The fact that no correlation was found between the DNA methylation levels of the selected genes and the telomere length in the control group is intriguing. First, we cannot preclude in the controls that other genes undergo changes in DNA methylation levels in relation to telomere length, since we have studied a very small number of genes. Second, these genes were found to be differentially methylated in meditation practitioners and meditation may be related to the maintenance of telomere length^12–16^. Thus, finding a relationship between methylation of these genes and telomere length is interesting and opens up the venue to further study these genes as targets of epigenetic changes in meditation practitioners.

As mention above, these genomic spots were found to be differentially methylated in meditators compared to controls in our previous work^35^. In the present study, DNA methylation levels at the *GPR31* gene were positively correlated to telomere length. *GPR31* (G protein-coupled receptor 31) encodes a high-affinity cell membrane receptor for 12-S-HETE, which is an arachidonic acid metabolite secreted by platelets and tumor cells that induces endothelial cell retraction and plays a role in extravasation and metastasis^62^. Notably, *GPR31* has been proposed as a target of oncology therapies since *GPR31* mediates *KRAS* (Kirsten rat sarcoma viral oncogene homolog) membrane association^35,63^, an important mechanism of tumorigenesis. More recently, *GPR31* was reported to underlie hepatic ischemia-reperfusion injury^64^.

In addition, DNA methylation levels at the *SERPINB9* gene were inversely related to telomere length. *SERPINB9* (Serpin B9) encodes a member of the serine protease inhibitor family, also known as serpins. *SERPINB9* inhibits the activity of the pro-apoptotic effector molecule granzyme B^65^ and therefore protects cytotoxic T-lymphocytes from apoptosis^66^. It is also expressed in accessory immune cells, such as dendritic cells, where it plays a role in presenting antigens^67^. *SERPINB9* could contribute to immune evasion in leukemia cells^68^ and to overcoming intracellular cytotoxicity in neuroectodermal tumors^69^. It has also been involved in autoimmune diseases^70^, such as celiac disease^71^. Interestingly, *SERPINB9* expression is reduced in atherosclerotic lesions^72^, and the axis *SERPINB9*/granzyme B has been proposed to modulate inflammation and insulin resistance in coronary atherosclerosis^68^. A previous report found S*ERPINB9* to be differentially methylated in blood cells of patients with abdominal aortic aneurysms^71^. S*ERPINB9* involvement in inflammatory pathways makes it biologically plausible the reported link with telomere length since inflammation is one of the postulated mechanisms to underlie the effect of meditation on improving human health^33,53^.

Still, the association of DNA methylation levels on those particular genes and telomere length is interesting even though the underlying mechanisms are far to be unraveled. It may be that these particular genes display a function on the general biology of telomeres that is still not known. Further investigations on ablation or overexpression of these genes in *in vitro* or *in vivo* models and their effect on telomere length would be of help to elucidate this point. On the other hand, the relationship between DNA methylation levels and mRNA expression levels for these genes is still not clear. For example, a report showed an inverse correlation between methylation and expression for *SERPINB9*^73^ while a direct correlation was found in other work^74^. In any case, as those genes were differentially methylated in long-term meditators, we could hypothesize that these particular genes may be a biological target of meditation. However, whether those genes are definitely involved in telomere biology can only be investigated by other type of experiments such as silencing or overexpressing them in cell or animal models. As for the mechanisms by which meditation changes DNA methylation at particular genes are still far to be understood.

**Figure 3:**
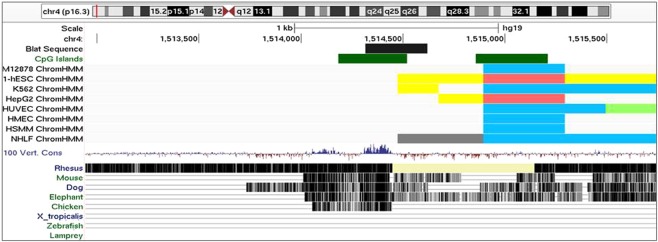
Genomic mapping of the intergenic subtelomeric region at the short arm of chr4. The graph was obtained from the UCSC Genome Browser and shows genomic position of the third DMR in long-term meditators compared to controls that was associated with telomere length. The black box represents the DMR (chr4:1514317–1514621, GRCh37/hg19 assembly). The green boxes represent CpG islands. Below, predicted functional elements are shown for each of eighth human cell lines explored by Chromatine imunoprecipitation (ChIP) combined with massively parallel DNA sequencing. Boxes represent insulators (blue), promoter regions (red), enhancers (yellow), and weak transcribed regions (light green). This track was obtained from Chromatin State Segmentation by HMM from ENCODE/Broad track shown at the UCSC Genome Browser. At the bottom, the conserved evolutionary conservation state across vertebrate species is shown for this region. The track was obtained from Vertebrate Multiz Alignment & Conservation (100 Species) at the UCSC Genome Browser.

Finally, telomere length inversely correlated with DNA methylation levels at a CpG island located in the subtelomeric region of the short arm of chromosome 4. This result is intriguing, as this locus is a gene desert, a region devoid of protein-coding genes. The differentially methylated CpG island is conserved across vertebrate species (Fig. 3). It seems to overlap an enhancer region in human embryonic stem cells (H1-hESCs) and is only 400 bp apart from an insulator region conserved across different cell lines. Further research will be needed to elucidate the role of this subtelomeric region on influencing telomere length maintenance.

However, this study is greatly exploratory and very limited in terms of not only statistical power but also regarding the possibility of establishing causal relationships. Judging by the effect sizes detected, significant relationships between age and telomere length might be observed in meditators when using larger samples. Nevertheless, we have observed that the effect size of the relationship between age and telomere length was drastically reduced in meditators when compared with non-meditator controls. In summary, this exploratory study will serve as a guide of future fully powered designs to clarify the true nature of all of these possible relationships.

## Conclusions

We had previously observed that differential DNA methylation changes in long-term meditators involved subtelomeric regions. Here, we show that DNA methylation levels at distinct subtelomeric regions encompassing *GPR31* and S*ERPINB9* genes are associated with telomere length only in the group of long-term meditators. However, we should be cautious about this result since it is above the threshold we used to correct for multiple-testing and therefore, it should be considered an exploratory study. In addition, age is no longer associated with telomere length in this group. The results of this exploratory study suggest that long-term meditation may be related to the age-related erosion of telomeres. Although the underlying mechanisms are not yet well understood, changes in DNA methylation arise as a factor that could contribute to this effect. Validation in an independent cohort would strengthen the results of the present study.

## Methods

### Participants

A group of long-term mindfulness meditators (MMs) (n = 17) was recruited from among members of the Spanish Association of Mindfulness and students enrolled in the Master of Mindfulness program at the University of Zaragoza. A comparison group of healthy controls (n = 17) was recruited from among healthy relatives and friends of the long-term MMs who had a similar lifestyle, and it was matched by gender, age (±2 years) and ethnic group. All the participants were Caucasian (European ancestry). Long-term MMs were required to have practiced meditation continuously for more than 10 years before the start of the study (including the previous 10 years) with a mean of at least 60 min/day of formal practice during the entire period. Information on participants´ meditation experience was also assessed, including the number of months that the participants had practiced meditation throughout their lifetime. The procedure and inclusion and exclusion criteria, along with socio-demographic, psychological, and health-related questionnaires, have been described elsewhere^35^.

### Telomere length measurement

From each subject, blood was obtained by venous puncture from the antecubital vein in sodium heparin tubes and processed within 24 h of collection to isolate peripheral blood mononuclear cells (PBMCs) using a Ficoll-Hypaque gradient. PBMC samples were rinsed in phosphate buffer solution, counted and resuspended at 10 million cells per milliliter in a freezing medium processed and stored in liquid nitrogen until further use. Telomere length assessment was performed as previously described^13^.

In brief, aliquots were thawed and diluted to plate 3.5 × 104 cells/well by quintuple in 384-well plates. Cells were fixed with methanol/acetic acid (3/1, *v*/*v*), treated with pepsin to digest the cytoplasm and the nuclei were then processed for *in situ* hybridization with a fluorescent Peptide Nucleic Ncid (PNA) probe that recognizes telomere repeats (sequence: Alexa488-OO-CCCTAACCCTAACCCTAA, Panagene). After several washing steps, as described by the standard DAPI incubation procedures for DNA staining, the wells were filled with mounting medium, and the plate was stored overnight at 4 °C.

Quantitative image acquisition and analysis were performed using the Opera High-Content Screening System (Perkin Elmer) with Acapella software, version 1.8 (Perkin Elmer, Waltham, MA, USA). Images were captured using a ×40 0.95 NA water immersion objective. UV and 488-nm excitation wavelengths were used to detect the DAPI and A488 signals, respectively. The results of the intensity of each foci identified were exported from the Acapella software (Perkin Elmer). Telomere length distribution and median telomere length were calculated with software from Life Length (www.lifelength.com). Samples with coefficients of variation lesser than 10% were accepted for the study.

### DNA methylation levels and differential methylation analysis

DNA was isolated from peripheral blood leukocytes by standardized methods^75^. DNA methylation data was generated using the Infinium HumanMethylation450 BeadChip array (Illumina, Inc., San Diego, CA, USA) at the Roswell Park Cancer Institute Genomics Shared Resource (Buffalo, NY, USA). 500 ng of genomic DNA from each sample was bisulfite treated and hybridized onto the BeadChip according to the manufacturer’s protocol. The percentage of methylation (β value) at each interrogated CpG site was calculated after quality control and normalization steps as described elsewhere^35^. In brief, image processing was carried out using the GenomeStudio Methylation Module (Illumina, Inc.). Background was corrected and adjustment was performed to avoid type I/II assay chemistry bias. To minimize technical variation and improve data quality we used the Dasen method^76^ as a normalization tool. Before performing differential methylation analysis, we removed probes overlapping common single nucleotide polymorphisms (SNPs) based on NCBI dbSNP Build 137 along with those probes classified as internal controls of the Illumina microarray. Additionally, probes located on the X and Y chromosomes were discarded along with probes that hybridized to multiple locations in the genome^77,78^. Probes that technically did not pass the Illumina quality threshold (1567 probes with beadcount <3 in >5% of samples and 535 probes having 1% of samples with a detection p-value > 0.05) were also removed. In the end, a total of 263 495 probes (representing CpG sites) were analyzed for differential methylation.

Differential methylation analysis was performed to identify differentially methylated regions (DMRs), defined as *loci* containing concordant and significant changes for neighboring CpGs (≥2 CpGs)^79^. We applied a Bioconductor package, *DMRcate*, that detect concordant and significant changes for neighboring CpGs by a kernel function to identify DMRs^80^. Methylation differences were prioritized by lowest p-values to ensure the most consistent DMRs between meditators and controls were included. These analyses identified sets of candidate *loci* with consistent differences in methylation in MM *versus* controls. An extended version of this section is included in Supplemental File 1.

### Mindfulness and psychological health-related variables

The Mindful Attention Awareness Scale (MAAS) is a 15-item scale used to measure awareness as a dispositional characteristic of paying attention to what is occurring in the present moment^81^. We used the Spanish version of the MAAS, which has displayed the appropriate psychometrics, with an internal consistency of α = 0.89^82^.

The Five Facet Mindfulness Questionnaire (FFMQ) is a 39-item questionnaire that evaluates five components of mindfulness: observing, describing, acting with awareness, non-judging of inner experience, and non-reactivity to inner experience^83^. The Spanish validated version of the FFMQ^84^, which has demonstrated adequate consistency values (α ≥ 0.80 in all the facets), was used.

The Hospital Anxiety and Depression Scale (HADS) is a 14-item questionnaire used to measure anxiety and depression^85^. The validity and reliability of the Spanish version of the HADS has shown to be adequate^86^, with internal consistency values in the corresponding subscales of anxiety and depression of α = 0.84 and α = 0.85, respectively.

The Connor-Davidson Resilience Scale (CD-RISC) is a 10-item scale used to measure resilience, defined as the psychological ability to successfully cope with adversity^87^. We used the Spanish validated version of the CD-RISC^88^, which has shown good psychometric characteristics (α = 0.85).

The Subjective Happiness Scale (SHS) is a 4-item scale used to measure subjective global happiness^89^. The Spanish version of the SHS^90^ was used, which has demonstrated good validity and also appropriate reliability values (α = 0.72).

The Self-Compassion Scale (SCS) is a 26-item scale that measures three facets of self-compassion (including negative aspects that are reverse coded): self-kindness, common humanity, and mindfulness (defined as the specific ability to keep emotions in balance when something is upsetting)^91^. We used the Spanish version of the SCS, with alpha values ranging from α = 0.72 to α = 0.79^92^.

The Satisfaction With Life Scale (SWLS) is a 5-item scale through which participants rate their overall satisfaction with their lives^93^. This questionnaire has been validated in Spanish^94^ with good validity and reliability parameters (α = 0.86).

The Experiential Avoidance Questionnaire (AAQ-II) is a 7-item scale that assesses one’s unwillingness to experience emotions and thoughts, and the inability to be in the present and engage in valued behavior when unwanted emotions/thoughts are present^95^. The Spanish translated and validated version of the AAQ-II^96^, which has presented adequate psychometrics (α = 0.93), was used.

### mRNA expression analysis by RT-qPCR

Total RNA was isolated from blood samples using RNeasy Lipid Tissue Mini kit (QIAGEN, Redwood City, CA, USA), following manufacturer’s instructions. Genomic DNA was removed with recombinant DNase (TURBO DNA-free™ Kit, Ambion, Inc., Austin, TX, USA). RNA integrity was checked by 1.25% agarose gel electrophoresis under denaturing conditions. Concentration and purity of RNA were both evaluated with NanoDrop spectrophotometer. Only RNA samples showing a minimum quality index (260 nm/280 nm absorbance ratios between 1.8 and 2.2 and 260 nm/230 nm absorbance ratios higher than 1.8) were included in the study. Complementary DNA (cDNA) was reverse transcribed from 1500 ng total RNA with SuperScript® III First-Strand Synthesis Reverse Transcriptase (Invitrogen, Carlsbad, CA, USA) after priming with oligo-d (T) and random primers. RT-qPCR reactions were performed in triplicate with Power SYBR Green PCR Master Mix (Invitrogen, Carlsbad, CA, USA) in a QuantStudio 12K Flex Real-Time PCR System (Applied Biosystems, Foster City, CA, USA). Sequences of primer pair were designed using Real Time PCR tool (IDT, Coralville, IA, USA). Relative expression level of genes mRNA in a particular sample was calculated as previously described [19] and the geometric mean of *ACTB* and *TBP* genes was used as the reference gene to normalize expression values.

### Statistical data analysis

The socio-demographic data and psychological health-related variables were described using either means and standard deviations (SDs), or frequencies and percentages, depending on their nature. Hypergeometric test (cumulative) was used to test for DMRs enrichment in subtelomeric regions. The comparisons between groups (MMs and healthy controls) in these variables were performed using the corresponding Student’s t and chi-squared tests. We also used the Student’s t test to compare the telomere length between groups, and we incorporated an ANCOVA to the same comparison to adjust for age. The effect size (ES) of differences was estimated by means of Cohen’s d. Values from 0.10 to 0.30 are considered to be small; from 0.31 to 0.50 intermediate; and 0.51 and higher, strong^97^. The level of relationships between age and telomere length, as well as between telomere length and DNA methylation levels, was assessed by means of Pearson’s r correlations separately for each group. We also calculated multiple regression models to control for age the relationships between telomere length and DNA methylation. Standardized regression coefficients (beta) were used to assess the individual contribution of DNA methylation and age in explaining telomere length, and the Wald test was used to evaluate the statistical significance of influences. Determination coefficients (R^2^) were used to evaluate the raw explanatory power of DNA methylation on the telomere length, while adjusted multiple determination coefficients (R^2^_y.12_) were used to observe the grouped explanatory power of age and DNA methylation. Their significance was assessed using analysis of variance. All of the tests used were bilateral, and the significance level was α < 0.05. As an alternative to multiple testing correction, we draw at random 1000 sub-samples of 14 not-DMRs and compute the proportion of such random sets that exhibit a lower number of correlations with telomere length (in the meditators group) than when considering the 14 meditation-related DMRs. This analysis was built with replacement and by using a set of 32830 non DMRs. SPSS-19 (IBM, Inc., USA) statistical software package was used. There were corrections for multiple comparisons but this study is of a highly exploratory nature^98^.

### Ethics approval and consent to participate

The study was approved by the Regional Ethics Committee of Aragon (number PI13/0056) and was conducted in accordance with the ethical standards of the 1964 Convention norms of Helsinki and later modifications. All of the participants provided their written informed consent before participating in the study.

## Supplementary information

Supplemental Information.

## References

[1] Demarzo MM (2014). Mindfulness-based stress reduction (MBSR) in perceived stress and quality of life: an open, uncontrolled study in a Brazilian healthy sample. Explore.

[2] Sharma M, Rush SE (2014). Mindfulness-based stress reduction as a stress management intervention for healthy individuals: a systematic review. Journal of evidence-based complementary & alternative medicine.

[3] Grossman P, Niemann L, Schmidt S, Walach H (2004). Mindfulness-based stress reduction and health benefits. A meta-analysis. Journal of psychosomatic research.

[4] Keng SL, Smoski MJ, Robins CJ (2011). Effects of mindfulness on psychological health: a review of empirical studies. Clinical psychology review.

[5] Rouleau CR, Garland SN, Carlson LE (2015). The impact of mindfulness-based interventions on symptom burden, positive psychological outcomes, and biomarkers in cancer patients. Cancer management and research.

[6] Moynihan JA (2013). Mindfulness-based stress reduction for older adults: effects on executive function, frontal alpha asymmetry and immune function. Neuropsychobiology.

[7] Zhang MF (2015). Effectiveness of Mindfulness-based Therapy for Reducing Anxiety and Depression in Patients With Cancer: A Meta-analysis. Medicine.

[8] Serpa JG, Taylor SL, Tillisch K (2014). Mindfulness-based stress reduction (MBSR) reduces anxiety, depression, and suicidal ideation in veterans. Medical care.

[9] Olivo EL (2009). Protection throughout the life span: the psychoneuroimmunologic impact of Indo-Tibetan meditative and yogic practices. Annals of the New York Academy of Sciences.

[10] Bushell WC (2009). Longevity: potential life span and health span enhancement through practice of the basic yoga meditation regimen. Annals of the New York Academy of Sciences.

[11] Jacobs TL (2011). Intensive meditation training, immune cell telomerase activity, and psychological mediators. Psychoneuroendocrinology.

[12] Conklin QA (2018). Insight meditation and telomere biology: The effects of intensive retreat and the moderating role of personality. Brain, behavior, and immunity.

[13] Alda M (2016). Zen meditation, Length of Telomeres, and the Role of Experiential Avoidance and Compassion. Mindfulness.

[14] Krishna BH, Keerthi GS, Kumar CK, Reddy NM (2015). Association of leukocyte telomere length with oxidative stress in yoga practitioners. Journal of clinical and diagnostic research: JCDR.

[15] Schutte NS, Malouff JM (2014). A meta-analytic review of the effects of mindfulness meditation on telomerase activity. Psychoneuroendocrinology.

[16] Thimmapuram J (2017). Effect of heartfulness meditation on burnout, emotional wellness, and telomere length in health care professionals. Journal of community hospital internal medicine perspectives.

[17] Martinez P, Blasco MA (2011). Telomeric and extra-telomeric roles for telomerase and the telomere-binding proteins. Nature reviews. Cancer.

[18] de Lange T (2002). Protection of mammalian telomeres. Oncogene.

[19] Blackburn EH (1991). Structure and function of telomeres. Nature.

[20] Bär Christian, Blasco Maria A. (2016). Telomeres and telomerase as therapeutic targets to prevent and treat age-related diseases. F1000Research.

[21] Greider CW, Blackburn EH (1985). Identification of a specific telomere terminal transferase activity in Tetrahymena extracts. Cell.

[22] Harley CB, Futcher AB, Greider CW (1990). Telomeres shorten during ageing of human fibroblasts. Nature.

[23] Hastie ND (1990). Telomere reduction in human colorectal carcinoma and with ageing. Nature.

[24] Lindsey J, McGill NI, Lindsey LA, Green DK, Cooke HJ (1991). *In vivo* loss of telomeric repeats with age in humans. Mutat Res.

[25] D’Mello MJ (2015). Association between shortened leukocyte telomere length and cardiometabolic outcomes: systematic review and meta-analysis. Circulation. Cardiovascular genetics.

[26] Haycock PC (2014). Leucocyte telomere length and risk of cardiovascular disease: systematic review and meta-analysis. BMJ (Clinical research ed.).

[27] Hagg S (2017). Short telomere length is associated with impaired cognitive performance in European ancestry cohorts. Translational psychiatry.

[28] Blasco MA (2007). Telomere length, stem cells and aging. Nature chemical biology.

[29] Wright WE, Shay JW (1992). Telomere positional effects and the regulation of cellular senescence. Trends Genet.

[30] Blasco MA (2005). Telomeres and human disease: ageing, cancer and beyond. Nature reviews. Genetics.

[31] Mather KA, Jorm AF, Parslow RA, Christensen H (2011). Is telomere length a biomarker of aging? A review. The journals of gerontology. Series A, Biological sciences and medical sciences.

[32] Muezzinler A, Zaineddin AK, Brenner H (2013). A systematic review of leukocyte telomere length and age in adults. Ageing research reviews.

[33] Rizvi S, Raza ST, Mahdi F (2014). Telomere length variations in aging and age-related diseases. Current aging science.

[34] Sanders JL, Newman AB (2013). Telomere length in epidemiology: a biomarker of aging, age-related disease, both, or neither?. Epidemiologic reviews.

[35] García-Campayo Javier, Puebla-Guedea Marta, Labarga Alberto, Urdánoz Amaya, Roldán Miren, Pulido Laura, de Morentin Xabier Martínez, Perdones-Montero Álvaro, Montero-Marín Jesús, Mendioroz Maite (2017). Epigenetic Response to Mindfulness in Peripheral Blood Leukocytes Involves Genes Linked to Common Human Diseases. Mindfulness.

[36] Buxton JL (2014). Human leukocyte telomere length is associated with DNA methylation levels in multiple subtelomeric and imprinted loci. Scientific reports.

[37] Yang J (2016). Tet Enzymes Regulate Telomere Maintenance and Chromosomal Stability of Mouse ESCs. Cell reports.

[38] Gonzalo S (2006). DNA methyltransferases control telomere length and telomere recombination in mammalian cells. Nature cell biology.

[39] Blasco MA (2007). The epigenetic regulation of mammalian telomeres. Nature reviews. Genetics.

[40] Benetti R, Garcia-Cao M, Blasco MA (2007). Telomere length regulates the epigenetic status of mammalian telomeres and subtelomeres. Nature genetics.

[41] Yehezkel S, Segev Y, Viegas-Pequignot E, Skorecki K, Selig S (2008). Hypomethylation of subtelomeric regions in ICF syndrome is associated with abnormally short telomeres and enhanced transcription from telomeric regions. Human molecular genetics.

[42] Toubiana S (2018). Subtelomeric methylation distinguishes between subtypes of Immunodeficiency, Centromeric instability and Facial anomalies syndrome. Human molecular genetics.

[43] Choudhury SR, Cui Y, Milton JR, Li J, Irudayaraj J (2015). Selective increase in subtelomeric DNA methylation: an epigenetic biomarker for malignant glioma. Clinical epigenetics.

[44] Oh BK, Um TH, Choi GH, Park YN (2011). Frequent changes in subtelomeric DNA methylation patterns and its relevance to telomere regulation during human hepatocarcinogenesis. International journal of cancer.

[45] Martinez P, Blasco MA (2017). Telomere-driven diseases and telomere-targeting therapies. J Cell Biol.

[46] Egusquiaguirre SP, Pedraz JL, Hernandez RM, Igartua M (2015). Emerging therapeutic approaches based on nanotechnology for the treatment of diseases associated with telomere dysfunction. Mini reviews in medicinal chemistry.

[47] van Ockenburg SL (2015). Stressful life events and leukocyte telomere attrition in adulthood: a prospective population-based cohort study. Psychological medicine.

[48] Verhoeven JE, van Oppen P, Puterman E, Elzinga B, Penninx BW (2015). The Association of Early and Recent Psychosocial Life Stress With Leukocyte Telomere Length. Psychosomatic medicine.

[49] Shalev I (2013). Stress and telomere biology: a lifespan perspective. Psychoneuroendocrinology.

[50] Epel ES (2004). Accelerated telomere shortening in response to life stress. Proc Natl Acad Sci USA.

[51] Denham J, O’Brien BJ, Charchar FJ (2016). Telomere Length Maintenance and Cardio-Metabolic Disease Prevention Through Exercise Training. Sports medicine.

[52] Ludlow AT, Ludlow LW, Roth SM (2013). Do telomeres adapt to physiological stress? Exploring the effect of exercise on telomere length and telomere-related proteins. BioMed research international.

[53] Arsenis NC, You T, Ogawa EF, Tinsley GM, Zuo L (2017). Physical activity and telomere length: Impact of aging and potential mechanisms of action. Oncotarget.

[54] Carlson LE (2015). Mindfulness-based cancer recovery and supportive-expressive therapy maintain telomere length relative to controls in distressed breast cancer survivors. Cancer.

[55] Rathore M, Abraham J (2018). Implication of Asana, Pranayama and Meditation on Telomere Stability. International journal of yoga.

[56] Valdes AM (2005). Obesity, cigarette smoking, and telomere length in women. Lancet.

[57] Babizhayev MA, Savel’yeva EL, Moskvina SN, Yegorov YE (2011). Telomere length is a biomarker of cumulative oxidative stress, biologic age, and an independent predictor of survival and therapeutic treatment requirement associated with smoking behavior. American journal of therapeutics.

[58] Michaels RR, Huber MJ, McCann DS (1976). Evaluation of transcendental meditation as a method of reducing stress. Science.

[59] Wenneberg SR (1997). A controlled study of the effects of the Transcendental Meditation program on cardiovascular reactivity and ambulatory blood pressure. Int J Neurosci.

[60] Bushell WC, Theise ND (2009). Toward a unified field of study: longevity, regeneration, and protection of health through meditation and related practices. Ann N Y Acad Sci.

[61] Alexander CN, Langer EJ, Newman RI, Chandler HM, Davies JL (1989). Transcendental meditation, mindfulness, and longevity: an experimental study with the elderly. J Pers Soc Psychol.

[62] Guo Y (2011). Identification of the orphan G protein-coupled receptor GPR31 as a receptor for 12-(S)-hydroxyeicosatetraenoic acid. J Biol Chem.

[63] Fehrenbacher N, Philips MR (2017). Targeting RAS - will GPR31 deliver us a new path forward?. Molecular & cellular oncology.

[64] Zhang XJ (2018). An ALOX12-12-HETE-GPR31 signaling axis is a key mediator of hepatic ischemia-reperfusion injury. Nature medicine.

[65] Sun J (1996). A cytosolic granzyme B inhibitor related to the viral apoptotic regulator cytokine response modifier A is present in cytotoxic lymphocytes. J Biol Chem.

[66] Bird CH (1998). Selective regulation of apoptosis: the cytotoxic lymphocyte serpin proteinase inhibitor 9 protects against granzyme B-mediated apoptosis without perturbing the Fas cell death pathway. Molecular and cellular biology.

[67] Mangan MS (2017). Serpinb9 is a marker of antigen cross-presenting dendritic cells. Molecular immunology.

[68] Fritsch K, Finke J, Grullich C (2013). Suppression of granzyme B activity and caspase-3 activation in leukaemia cells constitutively expressing the protease inhibitor 9. Annals of hematology.

[69] Vermeulen JF (2016). Pediatric Primitive Neuroectodermal Tumors of the Central Nervous System Differentially Express Granzyme Inhibitors. PLoS One.

[70] van der Burgh R (2016). Reduced serpinB9-mediated caspase-1 inhibition can contribute to autoinflammatory disease. Oncotarget.

[71] Pohjanen VM (2013). Decreased expression of protease inhibitor 9, a granzyme B inhibitor, in celiac disease: a potential mechanism in enterocyte destruction and villous atrophy. International journal of immunopathology and pharmacology.

[72] Hendel A (2012). Proteinase inhibitor 9 is reduced in human atherosclerotic lesion development. Cardiovascular pathology: the official journal of the Society for Cardiovascular Pathology.

[73] Li Z, Zhuang X, Zeng J, Tzeng CM (2017). Integrated Analysis of DNA Methylation and mRNA Expression Profiles to Identify Key Genes in Severe Oligozoospermia. Frontiers in physiology.

[74] Toghill BJ, Saratzis A, Freeman PJ, Sylvius N, Bown MJ (2018). SMYD2 promoter DNA methylation is associated with abdominal aortic aneurysm (AAA) and SMYD2 expression in vascular smooth muscle cells. Clinical epigenetics.

[75] Miller SA, Dykes DD, Polesky HF (1988). A simple salting out procedure for extracting DNA from human nucleated cells. Nucleic Acids Res.

[76] Pidsley Ruth, Y Wong Chloe C, Volta Manuela, Lunnon Katie, Mill Jonathan, Schalkwyk Leonard C (2013). A data-driven approach to preprocessing Illumina 450K methylation array data. BMC Genomics.

[77] Chen YA (2013). Discovery of cross-reactive probes and polymorphic CpGs in the Illumina Infinium HumanMethylation450 microarray. Epigenetics.

[78] Price ME (2013). Additional annotation enhances potential for biologically-relevant analysis of the Illumina Infinium HumanMethylation450 BeadChip array. Epigenetics & chromatin.

[79] Jaffe AE (2012). Bump hunting to identify differentially methylated regions in epigenetic epidemiology studies. International journal of epidemiology.

[80] Peters TJ (2015). *De novo* identification of differentially methylated regions in the human genome. Epigenetics Chromatin.

[81] Brown KW, Ryan RM (2003). The benefits of being present: mindfulness and its role in psychological well-being. Journal of personality and social psychology.

[82] Soler J (2012). Psychometric proprieties of Spanish version of Mindful Attention Awareness Scale (MAAS). Actas espanolas de psiquiatria.

[83] Baer RA, Smith GT, Hopkins J, Krietemeyer J, Toney L (2006). Using self-report assessment methods to explore facets of mindfulness. Assessment.

[84] Cebolla A (2012). Psychometric properties of the Spanish validation of the Five Facets of Mindfulness Questionnaire (FFMQ). The European Journal of Psychiatry.

[85] Zigmond AS, Snaith RP (1983). The hospital anxiety and depression scale. Acta psychiatrica Scandinavica.

[86] Herrero MJ (2003). A validation study of the hospital anxiety and depression scale (HADS) in a Spanish population. General hospital psychiatry.

[87] Campbell-Sills L, Stein MB (2007). Psychometric analysis and refinement of the Connor-davidson Resilience Scale (CD-RISC): Validation of a 10-item measure of resilience. Journal of traumatic stress.

[88] Notario-Pacheco B (2011). Reliability and validity of the Spanish version of the 10-item Connor-Davidson Resilience Scale (10-item CD-RISC) in young adults. Health and quality of life outcomes.

[89] Lyubomirsky SL, Lepper HS (1999). A Measure of Subjective Happiness: Preliminary Reliability and Construct Validation. Social Indicators Research..

[90] Extremera Natalio, Fernández-Berrocal Pablo (2013). The Subjective Happiness Scale: Translation and Preliminary Psychometric Evaluation of a Spanish Version. Social Indicators Research.

[91] Neff, K. D. Development and validation of a scale to measure self-compassion. *Self and Identity*, 223–250.

[92] Garcia-Campayo J (2014). Validation of the Spanish versions of the long (26 items) and short (12 items) forms of the Self-Compassion Scale (SCS). Health and quality of life outcomes.

[93] Diener E, Emmons RA, Larsen RJ, Griffin S (1985). The Satisfaction With Life Scale. Journal of personality assessment.

[94] Vazquez C, Duque A, Hervas G (2013). Satisfaction with life scale in a representative sample of Spanish adults: validation and normative data. The Spanish journal of psychology.

[95] Bond FW (2011). Preliminary psychometric properties of the Acceptance and Action Questionnaire-II: a revised measure of psychological inflexibility and experiential avoidance. Behavior therapy.

[96] Ruiz FJ, Langer Herrera AI, Luciano C, Cangas AJ, Beltran I (2013). Measuring experiential avoidance and psychological inflexibility: The Spanish version of the Acceptance and Action Questionnaire - II. Psicothema.

[97] Cohen, J. (Hillsdale, N. J.: L. Erlbaum Associates, 1988).

[98] Feise RJ (2002). Do multiple outcome measures require p-value adjustment?. BMC medical research methodology.

