# Supplementary material for: Telomere length correlates with subtelomeric DNA methylation in long-term mindfulness practitioners
Source: Sci Rep. 2020 Mar 12;10:4564. doi: 10.1038/s41598-020-61241-6 (PMC7067861; doi:10.1038/s41598-020-61241-6)
Supplement: Supplementary file 1 — Supplemental Information. [file 41598_2020_61241_MOESM1_ESM.pdf]

Telomere length correlates with subtelomeric DNA methylation in long-term mindfulness practitioners

Maite Mendioroz M.D., Ph.D[1, 2]*, Marta Puebla-Guedea[3], Jesús Montero-Marín Psy.D.[3], Amaya Urdánoz-Casado M.Sc[1]., Idoia Blanco-Luquin Ph.D[1], Miren Roldán[1], Alberto Labarga[4], Javier García-Campayo M.D., Ph.D[3, 5].

1. Neuroepigenetics Laboratory, Navarrabiomed Biomedical Research Center- Navarra Institute for Health Research (IdiSNA), Pamplona, Navarra, 31008, Spain
2. Department of Neurology, Complejo Hospitalario de Navarra, Pamplona, Navarra, 31008, Spain
3. Instituto de Investigación Sanitaria de Aragón. Red de Investigación en Atención Primaria (REDIAPP), Zaragoza, Spain.
4. Bioinformatics Unit, Navarrabiomed Biomedical Research Center - Navarra Institute for Health Research (IdiSNA), Pamplona, Navarra, 31008, Spain
5. Miguel Servet University Hospital, University of Zaragoza, Zaragoza, Spain

DNA methylation levels and differential methylation analysis

DNA was isolated from peripheral blood leukocytes by standardized methods(Miller, Dykes et al. 1988). DNA methylation data was generated using the Infinium HumanMethylation450 BeadChip array (Illumina, Inc., San Diego, CA, USA) at the Roswell Park Cancer Institute Genomics Shared Resource (Buffalo, NY, USA). 500 ng of genomic DNA from each sample was bisulfite treated and hybridized onto the BeadChip according to the manufacturer's protocol. A total of 485 577 cytosine positions were interrogated throughout the human genome, covering the 99% of RefSeq genes and 96% of CpG islands.

In order to minimize the potential bias introduced by batch effects, we performed samples-to-batch allocation using the OSAT tool (Yan et al., 2012). The percentage of methylation (β value) at each interrogated CpG site was calculated after quality control and normalization steps as described elsewhere (Javier García-Campayo, Marta Puebla-Guedea et al. 2017). In brief, microarray image processing was carried out using Genome Studio Methylation Module (v1.8.5) (Illumina, Inc.). Background was corrected and adjustment was performed to avoid type I/ II assay chemistry bias. To minimize technical variation and improve data quality we used the Dasen method (Pidsley, CC et al.) as a normalization tool.

Before performing differential methylation analysis, we removed probes overlapping common single nucleotide polymorphisms (SNPs) based on NCBI dbSNP Build 137 along with those probes classified as internal controls of the Illumina microarray. Additionally, probes located on the X and Y chromosomes were discarded along with probes that hybridized to multiple locations in the genome since cross-reactivity of

these regions can compromise true signal detection by the array (Chen, Lemire et al. 2013, Price, Cotton et al. 2013, Naeem, Wong et al. 2014). Probes that technically did not pass the Illumina quality threshold (1567 probes with beadcount <3 in > 5% of samples and 535 probes having 1% of samples with a detection p-value >0.05) were also removed. In the end, a total of 263 495 probes (representing CpG sites) were analyzed for differential methylation.

Differential methylation analysis was performed to identify differentially methylated regions (DMRs), defined as *loci* containing concordant and significant changes for neighboring CpGs (≥2 CpGs) (Jaffe, Murakami et al. 2012). We applied a Bioconductor package, *DMRcate*, that detect concordant and significant changes for neighboring CpGs to identify DMRs(Peters, Buckley et al. 2015). *DMRcate* is useful to identify and rank most differentially methylated regions across the genome based on tunable kernel smoothing of the differential methylation signal. The method is agnostic to both genomic annotation and local change in the direction of the DM signal, removes the bias incurred from irregularly spaced methylation sites, and assigns significance to each DMR called via comparison to a null model. Methylation differences were prioritized by lowest p-values to ensure the most consistent DMRs between meditators and controls were included. These analyses identified sets of candidate *loci* with consistent differences in methylation in MM *versus* controls.

REFERENCES

Chen, Y. A., M. Lemire, S. Choufani, D. T. Butcher, D. Grafodatskaya, B. W. Zanke, S. Gallinger, T. J. Hudson and R. Weksberg (2013). "Discovery of cross-reactive probes and polymorphic CpGs in the Illumina Infinium HumanMethylation450 microarray." Epigenetics **8**(2): 203-209.

Jaffe, A. E., P. Murakami, H. Lee, J. T. Leek, M. D. Fallin, A. P. Feinberg and R. A. Irizarry (2012). "Bump hunting to identify differentially methylated regions in epigenetic epidemiology studies." Int J Epidemiol **41**(1): 200-209.

Javier García-Campayo, Marta Puebla-Guedea, Alberto, Labarga, Amaya Urdánoz, Miren Roldán, Laura Pulido, X. M. de, Morentin, Álvaro Perdones-Montero, Jesús Montero-Marín and M. Mendioroz (2017). "Epigenetic response to mindfulness in peripheral blood leukocytes involves genes linked to common human diseases." Mindfulness.

Miller, S. A., D. D. Dykes and H. F. Polesky (1988). "A simple salting out procedure for extracting DNA from human nucleated cells." Nucleic Acids Res **16**(3): 1215.

Naeem, H., N. C. Wong, Z. Chatterton, M. K. Hong, J. S. Pedersen, N. M. Corcoran, C. M. Hovens and G. Macintyre (2014). "Reducing the risk of false discovery enabling identification of biologically significant genome-wide methylation status using the HumanMethylation450 array." BMC Genomics **15**: 51.

Peters, T. J., M. J. Buckley, A. L. Statham, R. Pidsley, K. Samaras, R. V Lord, S. J. Clark and P. L. Molloy (2015). "De novo identification of differentially methylated regions in the human genome." Epigenetics Chromatin **8**: 6.

Pidsley, R., Y. W. CC, M. Volta, K. Lunnon, J. Mill and L. C. Schalkwyk "A data-driven approach to preprocessing Illumina 450K methylation array data." BMC Genomics **14**: 293.

Price, M. E., A. M. Cotton, L. L. Lam, P. Farre, E. Emberly, C. J. Brown, W. P. Robinson and M. S. Kobor (2013). "Additional annotation enhances potential for biologically-relevant analysis of the Illumina Infinium HumanMethylation450 BeadChip array." Epigenetics Chromatin **6**(1): 4.
